# New insights into the genetic predisposition of brucellosis and its effect on the gut and vaginal microbiota in goats

**DOI:** 10.1038/s41598-023-46997-x

**Published:** 2023-11-16

**Authors:** Ahmed M. Sallam, Ibrahim Abou-souliman, Henry Reyer, Klaus Wimmers, Alaa Emara Rabee

**Affiliations:** 1 Animal and Poultry Breeding Department, Desert Research Center, Cairo, Egypt; 2 Research Institute for Farm Animal Biology (FBN), Wilhelm-Stahl-Allee 2, 18196 Dummerstorf, Germany; 3 Animal and Poultry Nutrition Department, Desert Research Center, Cairo, Egypt

**Keywords:** Genetics, Animal breeding, Genetic association study, Genetic markers, Genomics, Genotype, Heritable quantitative trait, Quantitative trait, Sequencing

## Abstract

Goats contribute significantly to the global food security and industry. They constitute a main supplier of meat and milk for large proportions of people in Egypt and worldwide. Brucellosis is a zoonotic infectious disease that causes a significant economic loss in animal production. A case–control genome-wide association analysis (GWAS) was conducted using the infectious status of the animal as a phenotype. The does that showed abortion during the last third period of pregnancy and which were positive to both rose bengal plate and serum tube agglutination tests, were considered as cases. Otherwise, they were considered as controls. All animals were genotyped using the Illumina 65KSNP BeadChip. Additionally, the diversity and composition of vaginal and fecal microbiota in cases and controls were investigated using PCR-amplicone sequencing of the V4 region of 16S rDNA. After applying quality control criteria, 35,818 markers and 66 does were available for the GWAS test. The GWAS revealed a significantly associated SNP (P = 5.01 × 10^–7^) located on Caprine chromosome 15 at 29 megabases. Four other markers surpassed the proposed threshold (P = 2.5 × 10^–5^). Additionally, fourteen genomic regions accounted for more than 0.1% of the variance explained by all genome windows. Corresponding markers were located within or in close vicinity to several candidate genes, such as *ARRB1, RELT, ATG16L2, IGSF21, UBR4, ULK1, DCN, MAPB1, NAIP, CD26, IFIH1, NDFIP2, DOK4, MAF, IL2RB, USP18, ARID5A, ZAP70, CNTN5, PIK3AP1, DNTT, BLNK,* and *NHLRC3.* These genes play important roles in the regulation of immune responses to the infections through several biological pathways. Similar vaginal bacterial community was observed in both cases and controls while the fecal bacterial composition and diversity differed between the groups (P < 0.05). Faeces from the control does showed a higher relative abundance of the phylum Bacteroidota compared to cases (P < 0.05), while the latter showed more Firmicutes, Spirochaetota, Planctomycetota, and Proteobacteria. On the genus level, the control does exhibited higher abundances of *Rikenellaceae RC9 gut group* and *Christensenellaceae R-7 group* (P < 0.05), while the infected does revealed higher *Bacteroides*, *Alistipes*, *and Prevotellaceae UCG-003* (P < 0.05). This information increases our understanding of the genetics of the susceptibility to *Brucella* in goats and may be useful in breeding programs and selection schemes that aim at controlling the disease in livestock.

## Introduction

Brucellosis is a bacterial infectious disease caused by various *Brucella* species^1^. It is categorized as a complex disease due to its wide range of hosts and the variable clinical signs^1^. It is a major zoonotic disease that infects domesticated and wild animals^2,3^, and is classified as a serious challenge and a neglected disease in developing countries, infecting about half a million people yearly^4,5^. Brucellosis is endemic and a highly contagious disease in the mediterranean countries, the middle east, south and central Asia and central and south America^4,6,7^. The disease causes significant economic losses to the animal production industry due to reducing animal productivity and fertility, fetuses’ losses, vaccination and veterinary care and culling costs^5,8–10^.

*Brucella melitensis* is the most causative prevalent species of the disease in small ruminants^11^. Animals become infected after the ingestion of contaminated milk, feed, water or grazing forage, close contact with infected animals, uterine secretions, or aborted foetuses^12–14^. The most notable clinical signs of the disease are abortion during the last third part of pregnancy, damages in male congenital tract with sterility, reduced fertility, reduced weight gain, a substantial decline in milk production and serious clinical complications to humans as well^5,15^. Humans acquire the disease through direct contact with infected animals or consumption of the contaminated animal products. Different *Brucella spp.* may infect humans^16^, however, *B. melitensis* transmitted from sheep and goats is the most contagious virulent pathogen^6,17,18^. However, data for the *Brucella* prevalence in Egypt are scarce, recent studies stated that the disease is endemic with high prevalence^19–21^. The prevalence of brucellosis in Egyptian goats ranged from 3.55^21^ to 11.3% according to different resources^22,23^ and causes a severe reduction in the profitability of animal production^18^.

Currently, there is no safe and effective *Brucella* vaccine available for use in humans^24^. Vaccines are available for animals but do not differentiate between infected and vaccinated animals on serological diagnostic tests^25^. Other tests, including molecular methods, have been proposed for the diagnosis of brucellosis in farm animals, although a combination of different methods appears most reliable for definitive identification so far^26^. It is well-known that genetics contribute significantly to complex traits^27^, including animal health and welfare^28,29^. Improvement in animal health through genetic selection is advantageous, because genetic gain is accumulative and permanent, as the polymorphisms introduced by breeding into a population can persist for many generations^30^. Identification of the host genetic predisposition to disease susceptibility could be a useful aid in programs that focus on management, screening, and culling of diseased animals^31^. It is worth to note that the vaginal and fecal microbiota has direct relationships to animal performance as it affects animal fertility^32^, and thus the profitability of animal farms. Therefore, exploring the vaginal and fecal microbiome could assist to predict the reproduction success and may be considered in selection programs^33,34^.

Few genetic variants and candidate genes have been identified to contribute to brucellosis in humans^35–38^ and livestock^39–43^. This suggests that there are individual variations in the degree of susceptibility or resistance to the disease^44^. Besides, understanding the changes in the vaginal and gut microbiota in case and control animals could improve their reproductive performance^32,45^. Limited information is available for the disease related traits and its effect on the vaginal and gut microbiome in livestock. The objective of this study was to scan animals’ genomes to identify genetic markers and candidate genes underlying susceptibility to brucellosis infection in Egyptian goats. Also, the effect of brucellosis infection on the diversity and composition of vaginal and fecal microbial community was considered. This may help in management system and controlling programs of the disease.

## Materials and methods

### Population and phenotype definition

All animal procedures included in the current study were approved by the animal breeding committee at the Desert Research Center (DRC) in Egypt. Along with the relevant ARRIVE (https://arriveguidelines.org/), all methods were performed in accordance with the relevant guidelines and regulations. A total number of 96 Damascus goats from two independent flocks located in the North-costal region of Egypt, were included in this study. The most obvious sign of brucellosis infection was recorded in the first flock (n = 76). More than half of the does have aborted at the last third period of pregnancy with retained placenta, while the remaining does have delivered their kids successfully. The second flock consisted of 20 does were included in the analysis as controls because they all gave successful birth, and they were negative to all the subsequent brucellosis serological tests. All the animals received a similar diet consisting of alfalfa hay (50%) and barley grains (50%). All the samples used in this study were collected during the first week after abortion or delivery.

Blood samples for each animal in the study were collected from the jugular vein in vacutainer tubes containing EDTA and other plain tubes without anticoagulant. Subsequently, serum samples were initially tested against *Brucella* infection using the Rose Bengal Plate test (RPPT)^46^. Samples that showed positive reaction to RPPT were tested using serum tube agglutination test (STAT)^47^ to confirm the infection. For RPPT, equal volumes (30 μL) of standardized *B. melitensis* antigen and test serum were mixed thoroughly. Any appearance of agglutination was recorded as a positive result. According to the degree of agglutination, positive samples were classified as weakly positive ( +) to strongly positive (+ +  + +). The samples in which agglutination was not observed within 4 min were assigned to be negative ( −) to *Brucella* infection. Using STAT, significant titers were those determined to be ≥ 1/80, which were considered positive to *Brucella* infection, while the 1/40 titers were considered negative. According to the World Organization of Animal Health protocol, RPPT and STAT tests were repeated for the same animals twice with a period of 2 weeks between the two tests to confirm the infection (case *vs* control). Additionally, another criterion was taken into account, which was the reproductive status of the animal (abortion vs birth).

Finally, animals were considered cases if they have aborted at the last third period of pregnancy and were positive to both RPPT and STAT tests. Otherwise, they were considered control and matched with cases on the flock and proximal birth date.

### Goat genotyping

Genomic DNA was extracted from the whole blood of each doe using a Puregen Core Genomic DNA extraction from blood Kit A (Qiagen®, Hilden, Germany) according to the manufacturer’s protocol. The quantity and quality of extracted DNA was assessed using Nanodrop spectrophotometer. High-quality DNA samples (≥ 50 ng/µL) were genotyped at the Research Institute for Farm Animal Biology, Dummerstorf, Germany, using the Illumina®inc. Goat_IGGC_65K_v2 Infinium HD SNP chip (Illumina, San Diego, CA, USA), which contains 59,727 SNPs in total, evenly distributed throughout the caprine genome using iScan Reader (Illumina). The ARS1 goat assembly was used as a reference genome in this study. Genotype calling was performed using GenomeStudio software (Illumina) according to the manufacturer’s protocols.

**Figure 1:**
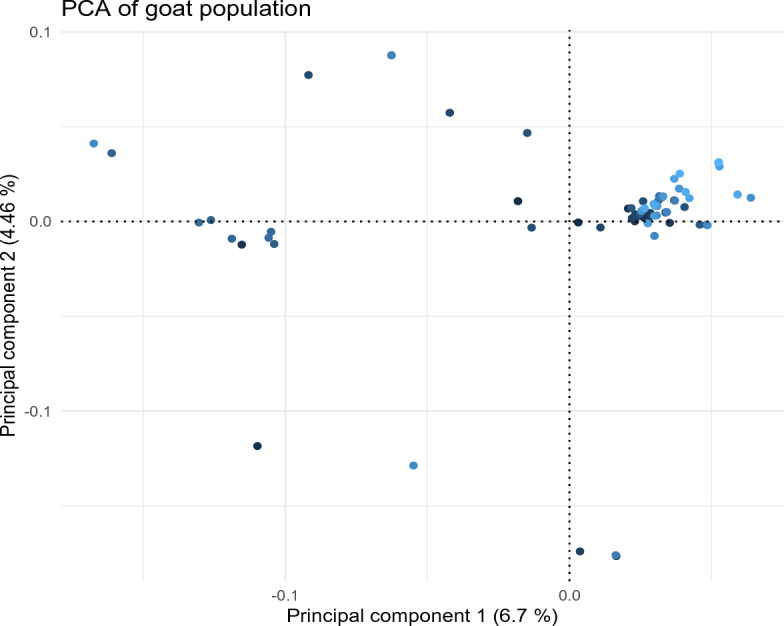
A Principal component analysis (PCA) plot representing the genetic landscape of 10 horse breeds extended across first and second components (PC1 and PC2) derived from eigen vectors and eigen values obtained from eigen decomposition of a genotypic (co)variance matrix between all individuals. Each color shows a different breed, and each point represents 1 sample.

A principal component analysis (PCA) was performed to illustrate the relationship between individuals using R software. The PC1 and PC2 (Fig. 1) that accounted for 6.7% and 4.64% of the genetic variation in the studied population, respectively, were only considered in the subsequent analysis. The genotyped population was filtered for quality in PLINK v1.9 software^48^, using the following parameters: (i) significant deviation from Hardy–Weinberg Equilibrium (HWE) *p* < 10^−6^; (ii) Minor Allele Frequency (MAF ≤ 0.01); (iii) genotype call rate < 0.99 for markers and < 90% for individuals. Furthermore, SNPs with unknown or identical chromosomal positions were also excluded from the subsequent analyses. A relatedness test was done using PLINK v1.9 to verify independence among the individuals. To avoid genomic inflation (i.e. the cryptic population substructure caused by the presence of closely related animals in the absence of pedigree information), pairwise identity-by-decent (IBD) was estimated for each pair of individuals in the population. Individuals of a pair that had a *pi-hat* value greater than 0.45 were considered as closely related, and thus were removed from the analysis.

### Genome-wide association analysis

Two statistical approaches based on fixation index (*F*_*st*_) and genome-wide association analysis were implemented in SNPRelate package in R^49^ and with the *–assoc* function in PLINK software v1.9, respectively. The Bonferroni correction of P-values was applied by accounting for the number of performed tests, suggesting a significance threshold of P < 5 × 10^–6^. To avoid false negative SNPs, another threshold of P < 5 × 10^–5^ was used following the Welcome Trust Case Control Consortium suggestion (WTCCC)^50^, which was also suggested by Duggal P. et al.^51^ as a conservative way to correct for the SNPs that are not truly independent. A quantile–quantile (Q-Q) plot of the observed P-values against expected P-values was created to evaluate the overall GWAS associations. A Manhattan plot of the negative common logarithm of SNP-specific P-values versus the chromosomal location was drawn using qqman package in R^52^.

### Variance components and heritability estimate

Due to the absence of pedigree data for animals included in this study, variance was proportioned, and SNP-based heritability (h^2^) was estimated based on the genomic information only using the Genome-wide complex trait analysis (GCTA) software^53^. First, restricted maximum likelihood (REML) analysis was performed until convergence of the likelihood ratio test (LRT). This option was followed by the option *‘–grm’* to estimate the variance explained by the SNPs that were used to estimate the genomic relationship matrix (GRM). Second, random effects were predicted by the best linear unbiased prediction (BLUP) method using *‘–reml-pred-rand’* to estimate the breeding value for each animal in this study attributed by the aggregative effect of the SNPs used to estimate the GRM followed by *‘–blup-snp’* flags implemented in GRMEL tool, which calculates the BLUP solutions for the SNP effects.

### Genetic variance explained by markers

The single-step GBLUP implemented in the BLUPF90 family^54^ was used to estimate the SNP effects from genomic estimated breeding values (GEBVs) of genotyped animals using the postGSf90 software of the BLUPF90 package^55^. SNP effects were calculated as: û = DZ' [ZDZ'] ^–1^ â_g_, where û is the vector of SNP effect; D is the diagonal matrix for weighting factors of the SNP effect; Z is the matrix of genotypes, and â_g_ is the vector of breeding values predicted for genotyped animals.The variance explained by each SNP was calculated as: σ^2^ = û^2^ 2p(1 − p), where û is the SNP effect described above and p is the allele frequency of the SNP^56^. The percentage of genetic variance explained by a window segment of 5 adjacent SNPs was calculated as: (Var (a_i_))/(σ^2^_a_) × 100%, where a_i_ is the genetic value of the *i*-th region that consists of 5 adjacent SNP and σ^2^_a_ is the total genetic variance^57^.

### Functional annotation of the significant SNPs

For each significant SNP (SNPs with a P-value equal to or exceeding the genome-wide threshold (P-value < 5 × 10^–5^) and the top 10 SNPs that accounted for the highest F_st_ values, a defined 2 Mb region (1 Mb on each side) was considered as a QTL interval. SNP locations reported in this paper are based on the genome version of *Capra hircus* available from the National Center for Biotechnology Information (ARS1, NCBI). Information on the SNPs and functional annotation of genes were obtained from BioMart at the Ensembl Genome Browser (http://www.ensembl.org/biomart)^58^. Functions of genes and encoded proteins were investigated using UniProt, OMIA (Online Mendelian Inheritance in Animals) and the GeneCards databases^59^.

### Vaginal and fecal microbiota

Based on the infection definition mentioned above, fecal and vaginal swab samples were collected from case and control animals included in the study (n = 8, each).

The vaginal samples were collected from each animal independently by inserting a sterile cotton swab into the vagina and rolled on the surface of vaginal epithelium for 30 s. The collected swabs were then stored at − 20 °C until subsequent analyses. The frozen swabs were thawed, re-suspended in 5 ml dissociation solution (0.1% Tween 80, 1% methanol, and 1% tertiary butanol (vol/vol), pH = 2), vortexed for 1 min and the supernatant solution was subsequently transferred to another sterile 50 ml tube. This step was further repeated twice and supernatants were collected. Then, the liquid was centrifuged at 12,000×*g* for 20 min to collect cell pellets that were used in DNA isolation using QIAamp DNA Stool Mini Kit (Qiagen, Hilden, Germany) according to the manufacturer’s instructions.

Fecal samples were collected from the rectum of the goats using clean gloves and samples were placed in sterilized 50 ml tubes that were frozen immediately at − 20 °C. Subsequently, 0.2 g fecal samples were used in DNA extraction using QIAamp DNA Stool Mini Kit (Qiagen, Hilden, Germany) according to the manufacturer’s instructions.

### PCR amplification, library sequencing, and data analysis

The quality and quantity of extracted nucleic acid were checked by Nanodrop spectrophotometer. The composition and diversity of microbial communities were studied by amplification of variable region V4 of the 16S rDNA gene by 515F and 926R primer sets using the following PCR conditions: 94 °C for 3 min; 35 cycles of 94 °C for 45 s, 50 °C for 60 s, and 72 °C for 90 s; and 72 °C for 10 min. The purified PCR-amplicons were sequenced using the Illumina MiSeq system at Integrated Microbiome Resource (IMR, Dalhousie University, Halifax, NS, Canada).

The generated paired-end (PE) Illumina raw sequences were analyzed in R software using the DADA2 pipeline^60^. The Fastq files of paired-end reads were demultiplexed and their quality was checked. Then after, the sequences were filtered, trimmed, and dereplicated followed by merging R1 and R2 reads together to get denoised sequences. The denoised sequences were subjected to removing the chimeras; then Amplicon Sequence Variants (ASVs) were obtained. Taxonomic assignment of ASVs was conducted using “*assign Taxonomy*” and “*addSpecies”* functions and microbial taxa were identified using SILVA reference database (version 138). Alpha diversity indices, including Chao1, Shannon, and InvSimpson were calculated. Moreover, beta diversity of microbial communities was calculated as principal coordinate analysis (PCoA) using bray–curtis dissimilarity. The differences in diversity indices and the relative abundances of bacterial phyla and genera were estimated by unpaired T-test. The raw sequence reads were deposited to SRA at https://www.ncbi.nlm.nih.gov/sra/PRJNA910086.

## Results

The quality control filtration process removed 15,051 SNPs and 10 animals, while 44,353 variants and 66 does passed QC criteria. The remaining SNPs were pruned for linkage disequilibrium using a window size of 1 Mb and a threshold of r^2^ > 0.5. Finally, 66 individuals (12 cases and 54 controls) genotyped at 35,818 SNPs were used in the subsequent GWAS analyses.

### Case–control GWAS

**Figure 2:**
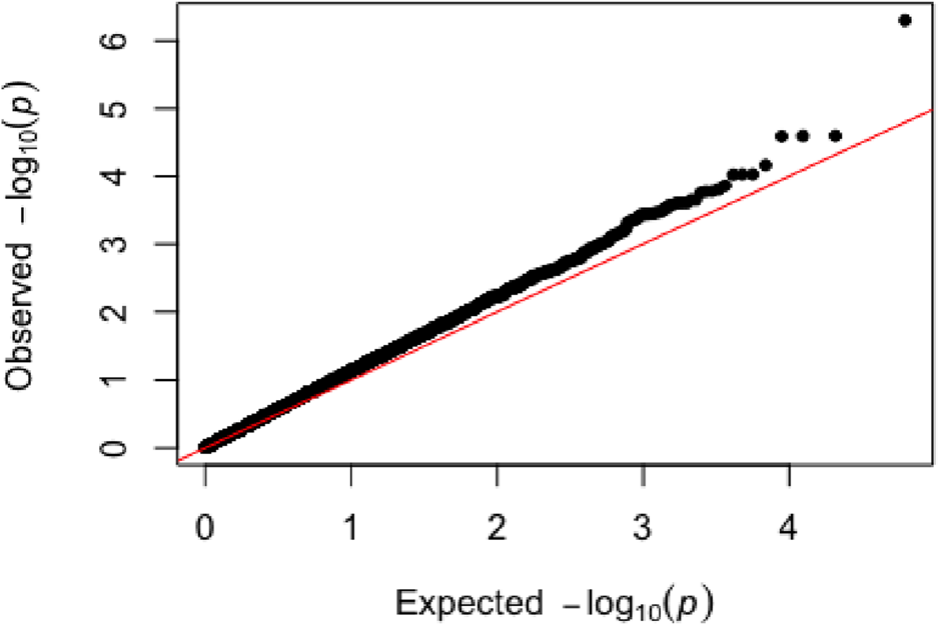
The Q-Q plot of the genome-wide association, where the -log10-transformed observed P-values (y-axis) are plotted against -log10-transformed expected P-values (x-axis).

**Figure 3:**
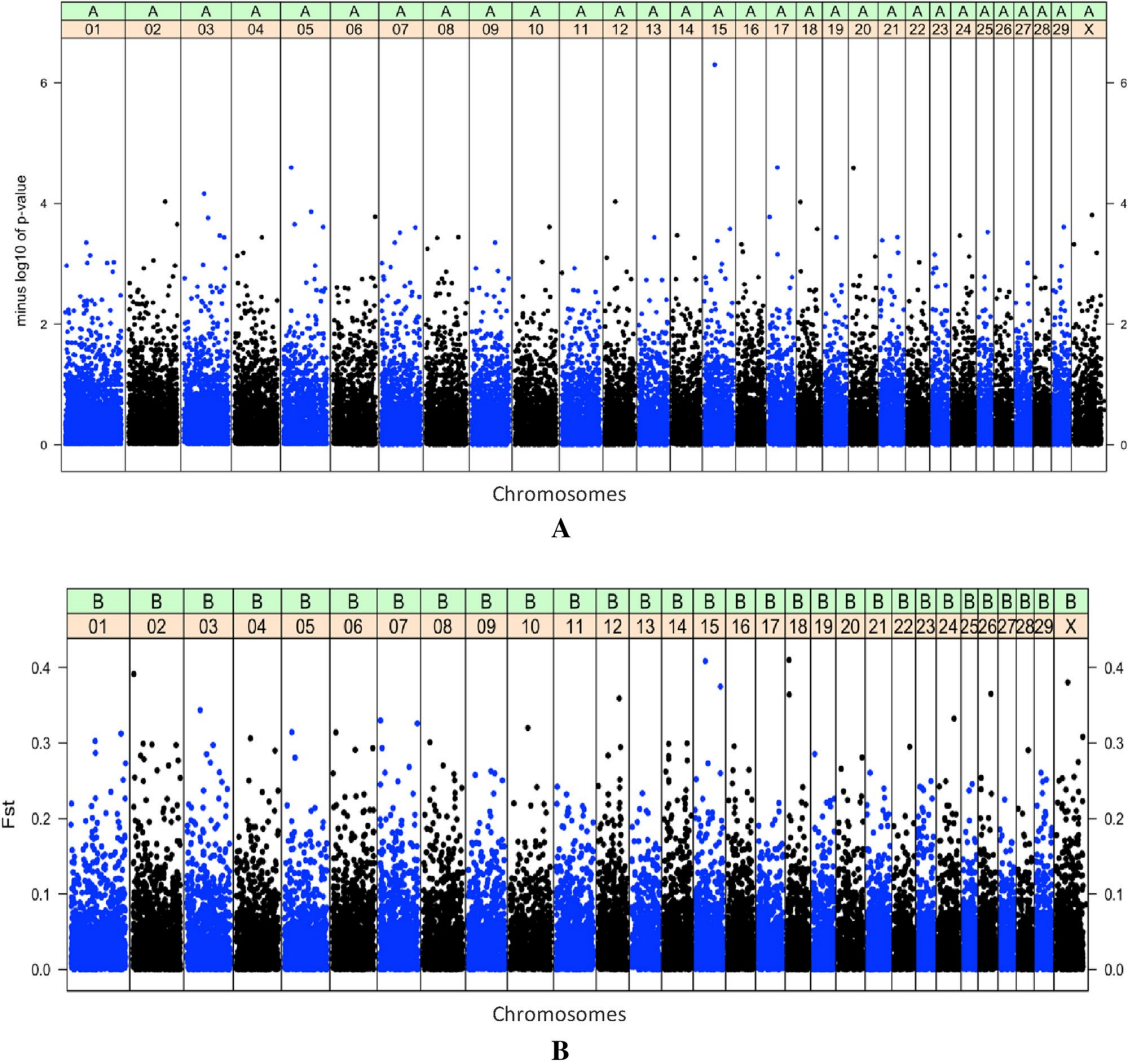
(**A**) Manhattan plot of genome wide association results susceptibility to brucellosis in Damascus goats. Each point represents a SNP. The solid dark blue line represents the threshold line and the second dark red solid line represents the genome-wide significance level for in -log10(P-value) scale in the y-axis and chromosomes are in the x-axis. (**B**) Manhattan plot of genome wide association results of susceptibility to brucellosis in Damascus goats. Each point represents a SNP. The solid dark blue line represents the threshold line and the second dark red solid line represents the genome-wide significance level for in Fst scale in the y-axis and chromosomes are in the x-axis.

Plotting the corrected P-values for most SNPs in the GWAS analysis exhibited a good correspondence to the expected P-values under the null hypothesis of no association, with the available number of SNPs indicating association with the trait under study (Fig. 2). The SNP array scans for brucellosis infection in Damascus goats (Fig. 3A) revealed that a SNP (*snp1723-scaffold1048-1212160*; P-value < 5.0 × 10^–7^) on chromosome 15 at 29 Mb surpassed the suggested genome-wide significance threshold level. The top 10 SNPs, some of which reach the WTCCC suggested threshold were located on chromosomes 17 (25 Mb), 5 (21 Mb), 20 (9 Mb), 3 (54 Mb), 2 (102 Mb), 12 (30 Mb), 18 (7 Mb), 5 (75 Mb) and 30 (39 Mb) (Table 1). The full details of the association test of all SNPs, MAF and SNP effect are located in the supplementary excel file. Among those, 3 SNPs were located within genes on chromosome 2 (102 Mb and 21 Mb) and chromosome 20 (9 Mb), while the remaining 7 SNPs were located in intergenic regions. Some of the significantly associated chromosomal regions harbored coding genes with biological roles contributing to T-cell signaling and controlling of the immune regulations and responses to infections.

**Table 1:** The 10 most-significant single nucleotide polymorphisms (SNP) and annotated genes for susceptibility to brucellosis in Damascus goats.

SNP_ID	Chr^1^	Location^2^	MAF^3^	Effect^4^	P-value	Nearest genes^5^
snp1723-scaffold1048-1212160	15	29,059,989	0.11	− 0.0001	5.01 × 10^–7^	ARRB1, RELT, ATG16L2
snp4664-scaffold1150-661894	17	25,373,675	0.22	− 0.0001	2.54 × 10^–5^	MMP17, ULK1, EP400
snp38477-scaffold486-4629898	5	21,122,756	0.13	− 0.0009	9.46 × 10^–5^	DCN
snp57399-scaffold913-2,774,950	20	9,455,389	0.14	− 0.0001	2.58 × 10^–5^	MAPB1, NAIP
snp10428-scaffold1373-362327	3	54,558,229	0.34	0.0001	5.64 × 10^–5^	IFI44L, IFI44, DNAJB4
snp20448-scaffold202-3614967	2	102,197,905	0.14	− 0.0005	6.86 × 10^–5^	DPP4, IFIH1, GCA, KCNH7
snp36088-scaffold431-8025178	12	30,589,940	0.066	− 0.0001	9.35 × 10^–5^	NDFIP2
18_7743438_RH-map	18	7,743,438	0.66	− 0.0001	9.35 × 10^–5^	DOK4, POLR2C, MAF
snp32384-scaffold368-1088126	5	75,087,691	0.26	− 0.0001	1.05 × 10^–5^	IL2RB, USP18

^1^Chromosome, ^2^Location in base pairs, ^3^Minor allele frequency, ^4^Estimated effect of the fitted allele, ^5^Based on the Ensembl database.

### *F*_*st*_ results

The top 10 SNPs that accounted for the highest F_st_ values in the current study (Fig. 3B) were observed on chromosomes 18 (7 Mb), 15 (29 Mb), 2 (2 Mb), 30 (25 Mb), 15 (72 Mb), 26 (34 Mb), 18 (7 Mb), 12 (63 Mb), 3 (36 Mb) and 24 (46 Mb) (Fig. 3) and accounted for F_st_ values of 0.41, 0.40, 0.39, 0.38, 0.37, 0.36, 0.36, 0.35, 0.34 and 0.33, respectively (Table 2). Information about all suggestive significant SNPs and the F_st_ estimates explained by each SNP are presented in the supplementary excel file. These identified chromosomal regions harbor candidate genes that encode proteins involved in the immunoglobulin superfamily and autophagy and pathways related to the innate immune system. A considerable overlapping was observed between the highest significantly associated genomic regions from GWAS and F_st_ approaches implemented in the current study. The common variants from both approaches were observed on Chr 18 (7 Mb) and Chr15 (29 Mb) and these genomic regions harbor important functional candidate genes for the disease.

**Table 2:** The top 10 single nucleotide polymorphisms (SNP) and annotated genes for susceptibility to brucellosis in Damascus goats based on *F*_*st*_ estimates.

SNP_ID	Chr^1^	Location^2^	MAF^3^	*F*_*st*_	Effect	Nearest genes^4^
snp21290-scaffold208-1536919	18	7,066,313	0.066	0.41	− 0.0001	DOK4, POLR2C, MAF
snp1723-scaffold1048-1212160	15	29,059,989	0.11	0.40	− 0.0001	ARRB1, RELT, ATG16L2
snp22295-scaffold220-1005859	2	2,609,074	0.48	0.39	–	IGSF21, UBR4,
snp4672-scaffold1151-52112	30	25,680,667	0.21	0.38	− 0.0001	–
snp13997-scaffold1553-193730	15	72,152,539	0.26	0.37	− 0.0002	CNTN5
snp17606-scaffold1829-380341	26	34,510,076	0.17	0.365	–	PIK3AP1, TLL2, DNTT, BLNK
18_7743438_RH-map	18	7,743,438	0.34	0.364	− 0.0001	DOK4, POLR2C, MAF
snp50111-scaffold717-1774945	12	63,381,016	0.09	0.359	− 0.0006	NHLRC3
snp15914-scaffold167-1388850	3	36,842,504	0.11	0.34	− 0.0001	–
snp40938-scaffold526-3777181	24	46,937,024	0.14	0.33	− 0.0001	EPG5, ZBTB7C, SMAD2

^1^Chromosome, ^2^Location in base pairs, ^3^Minor allele frequency, ^4^Based on the Ensembl database.

### SNP-based heritability

Genotypic (σ^2^g), residual (σ^2^e) and phenotypic (σ^2^p) variances and corresponding standard errors (SE) were estimated based on genomic information as 0.038 ± 0.039, 0.1 ± 0.04 and 0.14 ± 0.025, respectively. SNP-based h^2^ for susceptibility to brucellosis infection in goats based on genomic information was estimated as 0.26 ± 0.26.

### Genetic variance explained by markers

A genome window was considered to have a significant contribution to the genetic variance of the trait if it accounted for > 1%^61^. Accordingly, 14 genome windows with a total of 2.32% of genetic variance were identified. These windows were located on chromosomes 14 (17 Mb), 15 (72 Mb), 7 (40 Mb), 21 (46 Mb), 13 (48 Mb), 16 (43 Mb), 18 (15 Mb), 30 (22 Mb), 12 (0 Mb), 6 (84 Mb), 1 (66 Mb), 5 (20 Mb), 23 (4 Mb) and 30 (21 Mb) explaining about 0.47%, 0.43%, 0.2%, 0.12%, 0.12%, 0.12, 0.12%, 0.11%, 0.1%, 0.1%, 0.1%, 0.1%, 0.1% and 0.1% of the genetic variance, respectively (Table 3).

**Table 3:** The top genomic windows that accounted for more than 1% of the total genetic variance.

Chr^1^	Start^2^	End^3^	Genetic variance^4^	Candidate genes^5^
14	17,163,102	17,483,894	0.47	–
15	72,124,753	72,297,345	0.43	CNTN5
7	40,825,409	41,028,128	0.2	–
21	46,913,905	47,085,255	0.126	–
13	48,788,405	48,951,166	0.126	–
16	43,222,492	43,371,568	0.121	–
18	15,457,341	15,623,917	0.12	IL17-C and RNF166
30	22,087,163	22,217,172	0.116	–
12	708,771	900,439	0.1	–
6	84,058,501	84,262,013	0.1	TMBRSS11D
1	66,506,209	66,721,772	0.1	CD86, PARP9, DTX3L
5	20,141,506	20,329,430	0.1	–
23	4,816,235	5,017,478	0.1	–
30	21,770,505	21,926,068	0.1	–

^1^Chromosome, ^2^Starting location of the genomic window, ^3^Ending location, ^4^Genetic variance explained by the window (%), ^5^Based on the Ensembl database.

### Diversity and composition of vaginal bacteria

**Figure 4:**
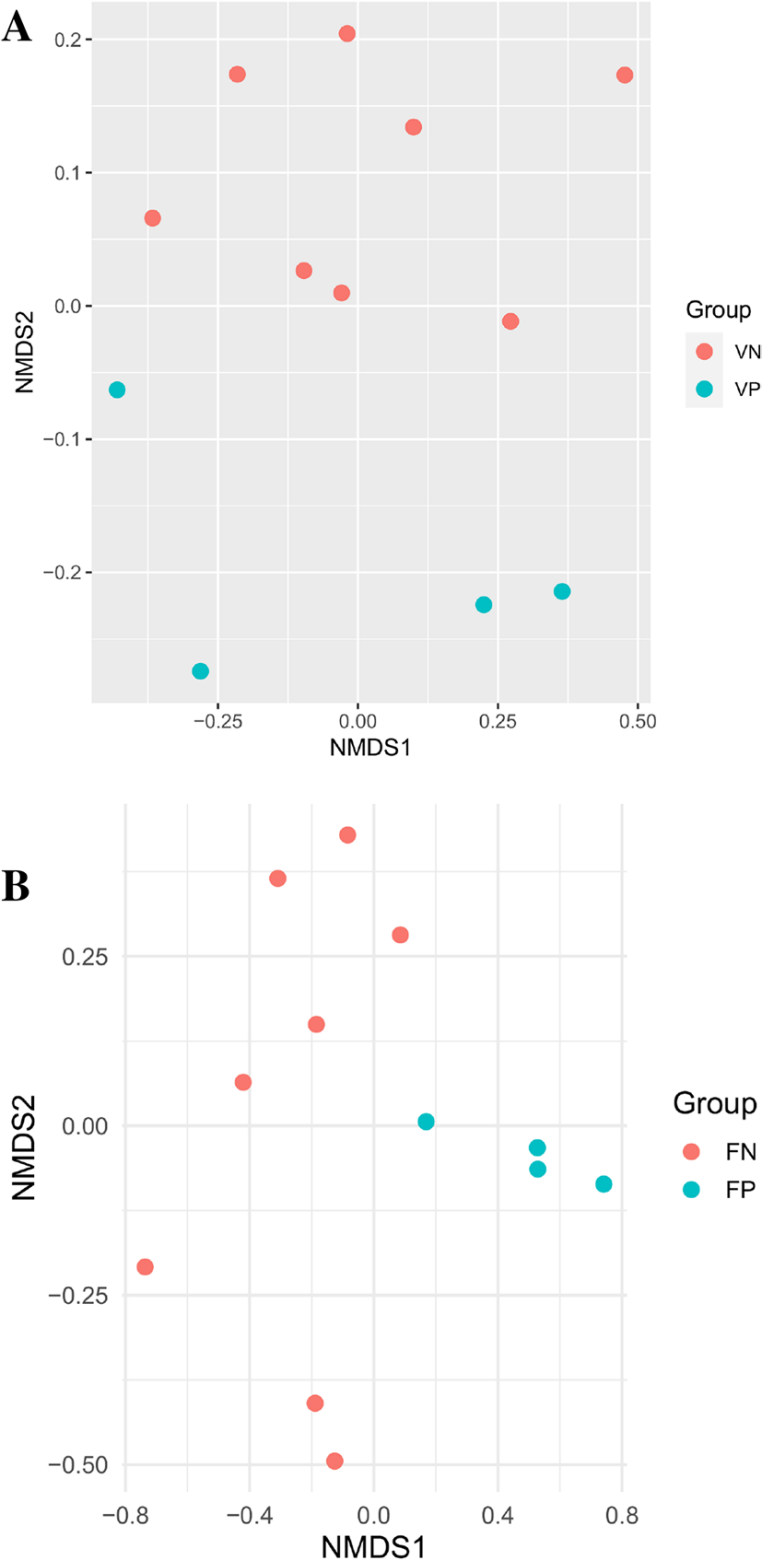
(**A**) Principal coordinates analysis of vaginal microbial communities of the non-infected (VN) and infected goats (VP) using Bray–Curtis dissimilarity. The red circles are for VN group and blue circles are for VP group. (**B**) Principal coordinates analysis of fecal microbial communities of the non-infected (FN) and infected goats (FP) using Bray–Curtis dissimilarity. The red circles are for FN group and blue circles are for FP group.

The sequencing failed in four vaginal DNA samples therefore fecal samples of the same animals were discarded from the study. The sequencing of 12 samples generated 1,035,511 high-quality sequence reads with a mean of 95,304 ± 11,841 (mean ± SE) reads per sample. The number of ASVs and alpha diversity indices, Chao1, Invsimpson, and Shannon, were greater in vaginal positive (VP) than vaginal negative (VN) group but this difference was not significant (P > 0.05) (Table 4). The PCoA analysis based on Bray Curtis metrics (Fig. 4A) showed that microbial communities of VN and VP were clustered separately. The analysis of the vaginal bacterial community in VN and VP revealed 15 bacterial phyla (Table 5). The bacterial community was dominated by phylum Proteobacteria and Firmicutes, which together constitute about 90% of the bacterial community. Bacterial phyla that represented 1–3.9% were Actinobacteriota and Bacteroidetes. Minor phyla that represented less than 1% were Spirochaetota, Planctomycetota, Deinococcota, Gemmatimonadota, Verrucomicrobiota, Acidobacteriota, Desulfobacterota, Myxococcota and Chloroflexi. Moreover, phylum Deferribacterota and Armatimonadota were detected exclusively in VP group (Table 5). Unclassified bacteria within vaginal bacteria represented more than 17% of the bacterial community (Table 6).

**Table 4:** The mean ± standard error (SE) of high-quality reads, number of Observed ASVs, Chao1, Shannon, and Inverse Simpson metrics of microbial communities of vaginal microbial communities of the brucellosis-infected (VP) and non-infected (VN) goats.

	VN		VP		Overall mean	SE	P value
	Mean	SE	Mean	SE			
Quality reads	85,656	11,312	114,602	27,550	95,304	11,841	0.25
Observed ASVs	397.87	34.81	558.75	99.36	451.5	43.98	0.07
Chao1	397.87	34.81	558.75	99.36	451.5	43.98	0.07
Shannon	4.26	0.14	4.4717	0.36	4.33	0.14	0.45
Invsimpsone	16.69	2.54	25.94	7.26	19.77	3.045	0.15

**Table 5:** The mean of relative abundance (%) of vaginal bacterial phyla in *Brucella* non-infected (VN) and infected goats (VP).

	VN		VP		Overall mean	SE	P value
	Mean	SE	Mean	SE			
Proteobacteria	49	3.04	45.31	9.12	47.65	3.60	0.64
Firmicutes	41.68	1.97	45.87	7.60	43.20	2.86	0.51
Actinobacteriota	1.59	0.29	1.50	0.53	1.55	0.25	0.06
Spirochaetota	0.35	0.12	0.76	0.56	0.50	0.21	0.87
Bacteroidetes	3.38	0.74	4.84	1.93	3.91	0.82	0.38
Planctomycetota	1.00	0.27	0.36	0.23	0.77	0.20	0.14
Deferribacterota	0	0	0.74	0.41	0	0	0
Deinococcota	0.18	0.071	0.10	0.05	0.15	0.048	0.46
Gemmatimonadota	0.26	0.18	0.07	0.018	0.19	0.11	0.45
Verrucomicrobiota	0.09	0.025	0.21	0.19	0.13	0.07	0.42
Acidobacteriota	0.06	0.03	0.06	0.02	0.06	0.02	0.84
Desulfobacterota	0.02	0.004	0.04	0.004	0.03	0.004	0.008
Armatimonadota	0	0	0.016	0.004	0	0	0
Myxococcota	0.02	0.005	0.013	0.004	0.017	0.003	0.39
Chloroflexi	0.02	0.002	0.006	0.002	0.016	0.003	0.001

**Table 6:** The mean of relative abundance (%) of vaginal dominant bacterial genera in *Brucella* non-infected (VN) and infected goats (VP).

	VN		VP		Mean	SE	P value
	Mean	SE	Mean	SE			
Phylum: Proteobacteria							
Burkholderia/Caballeronia/Paraburkholderia	20.41	2.98	13.93	2.47	18.25	2.27	0.19
Alcaligenes	2.87	0.74	6.20	3.38	3.97	1.22	0.22
Unclassified_Comamonadaceae	4.98	1.47	2.93	1.648	4.30	1.121	0.41
Halomonas	3.04	0.46	4.02	1.20	3.36	0.49	0.37
Ralstonia	0.25	0.25	3.80	2.67	1.43	0.96	0.08
Delftia	0.95	0.23	0.87	0.20	0.92	0.16	0.82
Pseudochrobactrum	0.15	0.06	1.19	0.65	0.50	0.25	0.045
Unclassified_Rhodobacteraceae	0.71	0.31	1.22	0.49	0.88	0.26	0.38
Pseudoxanthomonas	0.78	0.40	0.39	0.15	0.65	0.27	0.52
Bradyrhizobium	0.60	0.11	0.33	0.036	0.51	0.08	0.14
Paracoccus	1.50	0.30	0.72	0.14	1.24	0.23	0.11
Phylum: Firmicutes							
Lysinibacillus	2.96	1.14	1.94	0.67	2.62	0.78	0.56
Unclassified_Bacillales	3.36	0.67	2.95	1.07	3.22	0.54	0.74
Streptococcus	1.90	0.39	6.75	3.84	3.52	1.37	0.09
Unclassified_Aerococcaceae	4.32	1.85	1.16	1.10	3.27	1.32	0.28
Salinicoccus	0.82	0.19	2.21	1.49	1.28	0.51	0.21
Staphylococcus	1.64	0.31	2.85	1.46	2.04	0.51	0.29
UCG-005	4.65	1.20	2.2	1.04	3.83	0.91	0.22
Clostridium sensu stricto 1	0.89	0.34	1.33	0.58	1.04	0.29	0.51
Romboutsia	0.37	0.06	0.68	0.30	0.48	0.11	0.19
Unclassified_Oscillospirales	1.53	0.37	4.72	4.19	2.6	1.36	0.29
Planomicrobium	0.44	0.24	0.29	0.056	0.39	0.16	0.70
Lactobacillus	0.085	0.043	0.07	0.02	0.08	0.02	0.87
Phascolarctobacterium	0.50	0.20	0.25	0.14	0.42	0.14	0.44
Anoxybacillus	0.36	0.12	0.32	0.09	0.35	0.08	0.86
Exiguobacterium	0.44	0.10	0.16	0.035	0.35	0.078	0.09
Unclassified_Lachnospiraceae	2.16	1.54	1.08	0.55	1.80	1.03	0.64
Vagococcus	0.08	0.03	0.51	0.39	0.22	0.13	0.13
Unclassified_Oscillospiraceae	1.36	0.64	0.71	0.37	1.15	0.44	0.51
Christensenellaceae R-7 group	6.32	1.40	2.54	0.96	5.06	1.09	0.10
Phylum:Actinobacteriota							
Cutibacterium	0.48	0.15	0.22	0.048	0.39	0.11	0.28
Leifsonia	0.16	0.023	0.13	0.02	0.15	0.017	0.43
Corynebacterium	0.06	0.027	0.08	0.04	0.06	0.02	0.67
Micrococcus	0.06	0.04	0.03	0.03	0.05	0.03	0.63
Kocuria	0.08	0.02	0.06	0.015	0.08	0.014	0.53

Phylum Proteobacteria represented 47.65% of vaginal bacteria and was dominated by class Gammaproteobacteria and Alphaproteobacteria. On the genus level, phylum Proteobacteria was dominated by *Burkholderia-Caballeronia-Paraburkholderia*, *Alcaligenes*, *Halomonas*, *Ralstonia*, *Delftia*, *Pseudochrobactrum*, *Pseudoxanthomonas*, *Bradyrhizobium* and *Paracoccus*. Only genus *Pseudochrobactrum* was significantly higher (P < 0.05) in VP group compared to VN group. Additionally, unclassified bacteria in this phylum represented more than 5% (Table 6). The abundance of genus *Burkholderia-Caballeronia-Paraburkholderia* was declined from 20.4% in VN group to 13.93% in VP group. Genus *Alcaligenes* abundance was higher in VP group compared to VN.

Several bacterial genera were observed exclusively in a specific group of goats such as genus *Neisseria*, *Azohydromonas*, *Limnobacter*, *Pelagibacterium*, *Qipengyuania*, *Pseudorhizobium*, and *Cupriavidus* that were found in the VP group (Supplementary File 2). Also, genus *Reyranella*, *Phenylobacterium*, *Thermomonas*, *Paracoccus*, *Noviherbaspirillum*, *Conchiformibius*, *Devosia*, *Sphingomonas*, *Pseudorhodoferax*, *Neorhizobium*, *Shinella*, *Rubellimicrobium*, *Pseudoxanthobacter* and *Ellin6055* were observed in VN group (Supplementary File 2).

Phylum Firmicutes was the second largest phylum, representing 43.2% of the vaginal microbiome, and was classified mainly to class Bacilli and Clostridia (Tables 5 and 6). On the genus level, phylum Firmicutes was dominated by *Lysinibacillus*, *Streptococcus*, *Salinicoccus*, *Staphylococcus*, UCG-005, and *Christensenellaceae R-7 group*. The lactobacillus genus represented less than 0.1% and was higher abundance in VN group than VP group. Some bacterial genera that were observed in a specific group within Firmicutes, including *Romboutsia*, *Granulicatella*, *Gemella*, *Tumebacillus*, *Clostridium *sensu stricto* 6*, *Coprococcus*, *Solibacillus*, *Lachnoclostridium*, *Blautia*, *Epulopiscium*, and *Alkalibacterium* that were observed in VP group (Supplementary File 2). Also, genus *Aerococcus*, *Lachnospiraceae NK4B4 group*, *Weissella*, *Globicatella*, *Lachnospiraceae UCG-010*, *Granulicatella*, *Alkaliphilus*, *Fontibacillus*, *Dorea*, *Succiniclasticum*, *Veillonella*, *Trichococcus*, *Lachnospiraceae NK3A20 group*, *Marvinbryantia*, *Abiotrophia*, *Turicibacter*, *Pontibacter*, *Fibrisoma*, *Brachybacterium*, *Blastococcus*, and *Marmoricola* that were found exclusively in VN samples (Supplementary File 2). Phylum Desulfobacterota was significantly (P < 0.05) higher abundance in VP group whereas Chloroflexi was significantly higher abundance in VN group.

### Diversity and composition of fecal bacteria

The sequencing of 12 fecal samples resulted in 929,849 reads with a mean of 77,487 ± 8293 (mean ± SE) reads per sample. The number of ASVs as well as alpha diversity indices, Chao1, Inverse Simpson, and Shannon, were significantly (P < 0.05) higher in the cases (fecal positive, FP) than in the control (fecal negative, FN) (Table 7). The PCoA analysis based on Bray Curtis metrics (Fig. 4B) showed that microbial communities of FN and FP were clustered separately.

**Table 7:** The mean ± standard error (SE) of high-quality reads, number of Observed ASVs, Chao1, Shannon, and Inverse Simpson metrics of microbial communities of fecal microbial communities of the brucellosis-infected (FP) and non-infected (FN) goats.

	FN		FP		Mean	SE	P value
	Mean	SE	Mean	SE			
Reads	78,290	11,135	75,882	13,290	77,487	8293	0.89
Observed	446.12	32.48	843.25	68.45	578.5	63.71	0.0001
Chao1	469.4	37.12	922.56	86.82	620.46	73.60	0.0001
Shannon	5.15	0.07	5.88	0.066	5.40	0.11	0.0001
Invsimpsone	70.20	11.64	146.82	18.85	95.74	14.43	0.005

The fecal bacterial community was affiliated with 11 bacteria phyla; and two archaeal phyla, Halobacterota and Euryarchaeota that were observed only in FP group. The bacterial community was assigned mainly to Bacteroidotes and Firmicutes which represented together about 95%. The phylum Spirochaetota represented 1.7% of the bacterial community. Other minor bacterial phyla were Planctomycetota, Verrucomicrobiota, Desulfobacterota, Fibrobacterota, Proteobacteria, Actinobacteriota, and Cyanobacteria. Phylum Elusimicrobiota was observed only in the FP group (Table 8). Furthermore, unclassified sequence reads represented 0.27% of total sequenced reads.

**Table 8:** The mean of relative abundance (%) of fecal bacterial phyla in *Brucella* non-infected (FN) and infected goats (FP).

	FN		FP		Mean	SE	P value
	Mean	SE	Mean	SE			
Bacteroidota	28.53	1.96	20.30	2.63	25.79	1.90	0.03
Firmicutes	69.37	1.75	72.47	2.67	70.40	1.46	0.34
Spirochaetota	0.76	0.24	3.62	0.82	1.722	0.50	0.001
Planctomycetota	0.07	0.019	0.72	0.11	0.29	0.098	0.0001
Verrucomicrobiota	0.69	0.32	0.47	0.08	0.62	0.22	0.65
Halobacterota	0	0	0.25	0.054	0	0	0
Euryarchaeota	0	0	0.51	0.22	0	0	0
Desulfobacterota	0.08	0.02	0.15	0.018	0.10	0.02	0.06
Fibrobacterota	0.07	0.032	0.67	0.52	0.27	0.18	0.11
Unclassified	0.22	0.025	0.37	0.11	0.27	0.04	0.11
Proteobacteria	0.072	0.015	0.35	0.12	0.16	0.054	0.007
Actinobacteria	0.042	0.034	0.022	0.009	0.035	0.022	0.69
Cyanobacteria	0.07	0.024	0.039	0.018	0.06	0.017	0.43
Elusimicrobia			0.017	0.01			

The phylum Firmicutes dominated the bacterial community (70.41%) and was higher in FP group compared with FN group without significant difference. This phylum was dominated by *Christensenellaceae R-7 group* and *Lachnospiraceae AC2044 group* that were significantly declined in abundance (P < 0.05) in FP group compared to FN (Table 7). Genera *Phascolarctobacterium and NK4A214* group were significantly higher (P < 0.05) in FP group. Unclassified bacteria within Firmicutes represented 23.6% of the bacterial community (Table 9).

**Table 9:** The mean of relative abundance (%) of fecal dominant bacterial genera in *Brucella* non-infected (FN) and infected goats (FP).

	FN		FP		Mean	SE	P value
	Mean	SE	Mean	SE			
Phylum: Bacteroidota							
Rikenellaceae RC9 gut group	21.09	2.092	4.83	1.03	15.67	2.70	0.0001
Bacteroides	1.12	0.31	2.96	0.50	1.73	0.36	0.008
Family: M2PB4-65 termite group	0.89	0.82	0.26	0.09	0.68	0.548	0.60
Family F082	0.71	0.20	2.01	0.71	1.15	0.31	0.045
Alistipes	0.56	0.08	2.14	0.53	1.09	0.28	0.002
Prevotellaceae UCG-003	0.21	0.038	0.57	0.13	0.33	0.07	0.005
Family: Bacteroidales RF16 group	0.65	0.31	0.60	0.26	0.63	0.22	0.92
Prevotellaceae UCG-001	0.32	0.18	0.69	0.27	0.44	0.15	0.27
dgA-11 gut group	0.47	0.08	0.47	0.06	0.47	0.06	1
Prevotella	0.19	0.057	0.43	0.13	0.27	0.06	0.07
Phylum: Firmicutes							
UCG-005	7.79	0.29	7.03	0.65	7.54	0.29	0.24
UCG-002	1.86	0.34	3.42	0.43	2.38	0.34	0.02
Christensenellaceae R-7 group	22.45	2.19	11.12	1.70	18.67	2.21	0.007
Unclassified Clostridia	3.25	0.36	3.46	0.38	3.32	0.26	0.72
Lachnospiraceae AC2044 group	1.08	0.42	0.55	0.11	0.90	0.28	0.41
Unclassified Clostridia UCG-014	3.14	0.69	2.68	0.47	2.98	0.48	0.67
Phascolarctobacterium	0.24	0.06	1.25	0.39	0.58	0.19	0.005
Family: UCG-010	5.47	0.91	3.94	0.57	4.96	0.65	0.29
Lachnospiraceae UCG-010	0.64	0.17	1.013	0.18	0.76	0.135	0.20
UCG-009	0.92	0.21	1.01	0.13	0.95	0.14	0.80
NK4A214 group	1.06	0.14	2.07	0.15	1.39	0.17	0.001
Unclassified Oscillospiraceae	3.77	0.57	3.67	0.23	3.73	0.37	0.91
Unclassified Ruminococcaceae	2.56	0.77	1.80	0.07	2.30	0.51	0.51
Monoglobus	1.59	0.23	1.58	0.16	1.59	0.16	0.97
Unclassified Lachnospiraceae	4.07	0.49	5.13	0.51	4.42	0.38	0.21
Ruminococcus	1.42	0.32	1.58	0.44	1.47	0.24	0.78
Oscillibacter	0.37	0.05	0.326	0.06	0.36	0.037	0.58

The phylum Bacteroidetes, the second largest phylum in fecal bacterial community (25.79%), was significantly higher abundant in FN group than FP group (Table 8). On the genus level, this phylum was dominated by *Rikenellaceae RC9 gut* group that was declined in FP group. In addition, genera *Bacteroides*, *Alistipes*, and *Prevotellaceae UCG-003* were higher abundant in FP group (Table 9). Phylum Spirochaetota, Planctomycetota, and Proteobacteria were higher abundant in FP group compared with FN group (Table 8). Archaeal phylum Halobacterota was classified into genus *Methanocorpusculum*. Additionally, archaeal phylum Euryarchaeota was further classified to genera *Methanobrevibacter* and *Methanosphaera*.

## Discussion

The GWAS for brucellosis infection in Egyptian goats identified novel genetic markers, which may contribute to brucellosis susceptibility. It is important to understand the biological background of the candidate genes identified in the current study. The animal's response to the pathogens relies on the induction of multiple cell-mediated immune systems, such as the innate immune (e.g. *SLC11A1*, *TLR1*, and *TLR4*) and cytokine (e.g. *IFNGR1*, *IFNGR2*, *TNFA*) responses^62^. Hence, polymorphisms in the coding genes or genes of related pathways may contribute to the immune response capacity of the animal (i.e. disease tolerance or susceptibility). The genetic basis for variations in the animal response to the infectious diseases was previously reported in cattle suggesting the feasibility of genetic selection to improve resistance/tolerance to the disease^30^. In the current study, some of the significantly identified chromosomal regions harbor genes with biological roles in the innate immune response of the animal. For instance, the *snp57399-scaffold913-2774950* (*rs268288924*) on goat chromosome 20 was located in the intronic region of *MAP1B* (microtubule associated protein 1B) gene, which acts as a positive cofactor in DAPK1 (death associated protein kinase)-mediated autophagic vesicle formation and membrane blebbing^63^. The *snp20448-scaffold202-3614967* (*rs268252959*) on chromosome 2 mapped to the intronic region of *KCNH7* (potassium voltage-gated channel subfamily H member 7) gene. This gene was identified in the plasma membrane as a potassium channel^64^ to be involved in multiple cellular processes affect the immune system.

Importantly, three potential candidate genes were located within 1 Mb window of the most significant associated SNP on chromosome 15 (*snp1723-scaffold1048-1212160*, P = 5.01 × 10^–7^): (1) the arrestin beta 1 (*ARRB1*) gene, which is involved in Toll-like and IL-1 receptors signaling. This gene was found to be highly expressed in peripheral blood leukocytes and plays a major role in regulating receptor-mediated immune functions^65,66^. (2) The RELT-TNF receptor (*RELT*) gene, which activates the NF-kappaB pathway and binds TNF receptor-associated factor 1 (*TRAF1*). This receptor is capable of stimulating T-cell proliferation in the presence of CD3 signaling, which suggests its regulatory role in immune response^67^. (3) The autophagy related 16 like (*ATG16L2*) gene, which is involved in biological pathways related to autophagy. In agreement, several immunity-related genes have been identified for resistance/susceptibility to brucellosis infection in humans, such as IL-17^35^, IFN-R1^36^, TGF-β1^37^ and IL6 and IL10^38^. In contrast, four alleles in the *TLR5* gene were identified to be correlated with a seroprevalence of brucellosis in the Saanen goats^43^, as well as the innate immunity genes in cattle^68^. A haplotype in the *PTPRT* gene was associated with resistance to *Brucella* infection in Argentinian goats^41^. Associations of *TNF* rs668920841 and *INRA111* polymorphisms with caprine brucellosis was also reported^69^. Additionally, variability at the SLC11A1 locus has been linked to resistance to brucellosis in Algerian goat^70^. Few genetic variants and candidate genes have been associated with antibody response in feral swine infected with brucellosis^42^.

Likewise, other significant SNPs were located in intergenic regions of important candidate genes. For instance, *ULK1* (Unc-51 Like autophagy activating kinase 1) gene (CHR17, 25 Mb), which is involved in several processes including autophagosome assembly^71^. The *IFI44L* and *IFI44* (interferon induced proteins) genes at CHR3 (54 Mb) play a critical role in antiviral and antibacterial activity^72^. They promote macrophage differentiation and facilitate inflammatory cytokine secretions in the immune response to bacterial infection. The *snp20448-scaffold202-3614967* (P = 6.86 × 10^–5^) on chromosome 2 was in close proximity to three candidate genes (Dipeptidyl Peptidase; *DPP4*, also known as *CD26*, interferon induced with helicase C domain; *IFIH1* and Grancalcin; *GCA*), which are involved in the immune regulation and response to infections^73,74^. Similarly, *snp21290-scaffold208-1536919* on chromosome 18 at 70 Mb indicated two potential candidate genes: docking protein (*DOK4*) and transcription factor (*MAF*), which are involved in the regulation of the immune response induced by T-cells and activation of the expression of IL4 in T helper 2 cells^75,76^. The window at 75 Mb on chromosome 5 harbored *snp32384-scaffold368-1088126* indicating the interleukin 2 receptor subunit beta (*IL2RB*) gene, which is involved in IL2 and T cell-mediated immune responses^62^, and the ubiquitin specific peptidase (*USP18*) gene, which is involved in regulation of the inflammatory response triggered by type I interferon^77^.

An interesting genomic region on chromosome 26 (34 Mb) included 3 potential candidate genes that are involved in the immune system: (1) Phosphoinositide-3-Kinase Adaptor Protein (*PIK3AP1*) gene that contributes to B-cell development, activation of PI3K in natural killer cells and TLR signaling pathways, (2) DNA Nucleotidylexotransferase (*DNTT*) gene, which encodes a protein expressed in malignant pre-B and pre-T lymphocytes during early differentiation^78^, and (3) B Cell Linker (*BLNK*) gene that encodes a cytoplasmic linker or adaptor protein and plays a critical role in B-cell development^79^. Further chromosomal regions were identified on chromosomes 5 (72 MB), 12 (63 Mb) and 24 (46 Mb) comprising contactin (*CNTN5*), NHL repeat containing (*NHLRC3*) and ectopic P-Granules 5 autophagy tethering factor (*EPG5*) genes that encode proteins involved in the immunoglobulin superfamily^80^, autophagy and pathways related to the innate immune system^81^. A considerable overlap was observed between GWAS and F_st_ approaches pointing to chromosomes 18 (7 Mb) and 15 (29 Mb) with important candidate genes for the disease as shown above.

Comparison between our GWAS results and previous reports is difficult due to scarcity of studies addressing the genetic basis of brucellosis in livestock. Additionally, in case of infected animals, the availability of well-defined adequate phenotypes to clearly diagnose the disease are still challenging^27^. Accordingly, reanalyzing the data with additional phenotypes followed by fine mapping of positional candidate genes may confirm our findings and help to identifying novel candidate genes for brucellosis susceptibility in livestock.

SNP heritability measures the proportion of phenotypic variance explained by all markers without pedigree^82^. Such type of genomic-based heritability estimates for brucellosis infection in livestock was not reported yet. The pedigree-based h^2^ estimate was 0.33 for brucellosis infection in Awassi sheep^83^ was higher than estimated in our study in Damascus goats. This may be because of using different estimation methods, i.e. pedigree-based and genomic-based, respectively. However, the reasonable h^2^ estimated here means that SNP information captured most of the variance between individuals in context of the studied traits^84^. Comparably, quite similar estimates (h^2^ = 0.03–0.28) using either pedigree or genomic information was reported for paratuberculosis in dairy cattle^85^. This suggests that brucellosis infection may be heritable and could respond well to genetic improvement programs.

Quantitative traits may be affected by a few genes with large or modest effects plus genes with small effects, otherwise, they are affected by many genes with small effects^86^. In our study, we identified 14 genome windows each explained more than 0.1% of the total genetic variance and all together explained 2.32% of the genetic variance. These regions harbored genes of immunity, which may be suitable candidates for the susceptibility to brucellosis. Interestingly, the genome window located on chromosome 18 (15 Mb) harbored interleukin 17C (*IL17-C*) and ring finger protein 166 (*RNF166*) genes that play a crucial role in the innate immunity^87^ and autophagy^87^, respectively. Likewise, the genomic window on chromosome 1 (66 Mb) harbored *CD86*, poly (ADP-ribose) polymerase family (*PARP9*), and deltex E3 ubiquitin ligase (*DTX3L*) candidate genes, which are involved in the immune response^88,89^. Corresponding to *Fst* results, the region at 72 Mb on chromosome 15 that harbors the *CNTN5* gene was identified here to explain 0.43% of genetic variance. This suggests that this region may plays an important role in the disease susceptibility and may be worth to be involved in further investigations. Otherwise, none of the top significant SNPs were reported in the top genome windows that explain > 1% of phenotypic variance, suggesting that the trait is likely controlled by a multiple SNPs with small to medium effects^85^.

The immune system has a complex bidirectional relationship with the microbiome of the organism. The diversity of the fecal microbial community showed a crucial association with variations in the immune response, which in turn may alter the microbiome^90^. This is due to the direct effects of the intestinal microbiota on diet fermentation and animal feed efficiency^91,92^. Genetic polymorphisms were identified to influence the microbiome in humans. Noteworthy, the vaginal and fecal microbiome is significantly associated with animal performance^32^. For instance, it was reported that dysbiosis in the vaginal microbiome causes inflammation and declines reproductive efficiency^34,91–93^. In goats, no information is available on the vaginal and fecal microbiome and their relations with reproductive diseases such as brucellosis.

Reproductive disorders increase the microbial diversity in the reproductive tract^94^. This is consistent with our findings in the vaginal and fecal microbial communities and supported by the results of PCoA. In agreement, Lui et al.^95^ showed that the vaginal microbiome of aborted women showed higher diversity compared to healthy women. Additionally, the vaginal microbiome in this study was dominated by phylum Firmicutes and Proteobacteria, which agrees with previous studies on cattle^32^ and sheep^34^. In this study, the vaginal bacteria were dominated by microbes assigned to genera covering *Burkholderia, Caballeronia* and *Paraburkholderia*, which were lower abundant in the infected does compared to the non-infected. Consistently, the abundance of these genera was declined in the aborted women patients with recurrent spontaneous abortion (RSA)^96^. Moreover, corresponding species were observed in the semen microbiome and showed a positive correlation with the healthy status of the genital tract^97^. Infected animals showed a higher relative abundance of the genus *Halomonas*, which is a pathogen bacterium^95^. Genus *Alcaligenes* was higher abundant in the infected does which agrees with the pathogenic attributes assigned to species of this genus^98^. Furthermore, *Alcaligenes* is a drug-resistant genus and susceptible to a specific combination of antibiotics^98,99^. These findings highlight the importance of investigating the vaginal microbiome in animals with reproductive disorders to characterise the microbial milieu and identify the best suitable antimicrobial treatment. Genera *Streptococcus*, *Salinicoccus* and *Staphylococcus* cause vaginal disorders and abortion, which may explain their higher proportion in the infected does^32,100,101^. *Staphylococcus* was previously observed in ewes^34^ and dominated the vaginal microbiome of goat in estrus synchronization^33^. Unlike Human vaginal microbiome, *Lactobacillus* represented a small proportion in the goats’ vaginal microbiome^102^. Importantly, some of bacterial genera found exclusively in the infected does are pathogens (e.g. genus *Neisseria*), which may causes sexually transmitted diseases^103^.

The differences in the diversity and structure of fecal microbiome were significant between infected and non-infected animals. Also, the bacterial community in fecal samples was dominated by phylum Firmicutes and Bacteroidetes, which agrees with previous studies on fecal microbiota in goats and cattle^32,104^. Most of bacteroidetes’ members are specialized in the degradation of lignocellulose and soluble polysaccharides in the animal gut^105^, which may be a reason for decreasing the dietary fiber digestion in the infected animals. The dominant genera in the fecal microbiome were *Rikenellaceae RC9 gut group* and *Christensenellaceae R-7 group*, which is consistent with previous studies on goat fecal microbiome^104^ and the rumen microbiome of cattle^92^. These genera showed higher relative abundances in the non-infected animals. Members of family Christensenellaceae were related to a healthy phenotype in humans^106^. *Christensenellaceae R-7 group* abundance was positively correlated with animal health, feed efficiency, animal’s body mass index, rumen digestion and absorption of nutrients, fiber digestion, and protein metabolism; and this genus produces acetic and butyric acids^91,92^. Moreover, *Rikenellaceae RC9 gut group* plays a critical role in the digestion of crude fiber^92,105^. Accordingly, reproductive disorders could impair animal performance and gut fermentation. Specifically, fiberolytic bacteria not only digest dietary fiber but some also represent a barrier against pathogens^107^.

### Study limitations and future perspectives

Generally, identifying the causative variants for a specific disease is challenging^108^. Despite GWAS highlights signals in the entire genome that help to understand the biology of disease infection, they do not pinpoint the causative variants. There are many limitations of complex traits (e.g. disease susceptibility) analysis with GWAS. The small sample size, low or missing heritability^109^ and the phenotype definition (e.g. examining different stages of the disease progression) are of these limitations^110^. Therefore, it is difficult to identify SNPs with larger effects and there is a lot of discrepancies between GWAS results^111^. Besides, the identified variants do not capture most of the additive genetic variations due to the disease. However, these investigations are important to clarify the biological basis of disease, providing information about the mechanisms underlying the disease process^83^. In the current study, the small sample size and identification of the infectious status of brucellosis based on serological test only are limiting our results. Future studies with larger sample size are needed to confirm the identified SNPs that arise from a particular biological pathway. Alongside, combining multiple datasets may increase the statistical power and exhibit the overlapped chromosomal regions that was marginally identified in the previous analysis^112^. This may also encompass interdisciplinary molecular approaches, such as microbiology and PCR-based identification of the causative agent with different isolates to classify and identify the virulent strain of the disease^113^.

## Conclusion

Lack of enough information concerning the genetics of brucellosis in livestock is a real barrier towards animal welfare, diagnosis, vaccination, and management of the disease. The previous studies pointed out polymorphisms in immunity-related genes as putative candidate genes for disease susceptibility in humans and livestock. Accordingly, the identified variants and candidate genes from our GWAS analysis may contribute to the phenotypic variations between cases and controls of Brucella infection. This included chromosome 15 (29 Mb; *ARRB1, RELT* and *ATG16L2*), 20 (9 Mb; *MAP1B*), 2 (102 Mb; *KCNH7*), 17 (25 Mb, *ULK1*), 3 (54 Mb; *IFI44L* and *IFI44)*, 18 (7 Mb; *DOK4* and *MAF*), 5 (75 Mb; *IL2RB)* and 26 (34 Mb; *PIK3AP1, DNTT, BLNK*) as the most important genomic regions potentially contributing to the incidence of brucellosis in the examined goat population. Some of the identified QTLs were overlapped between F_st_ and GWAS approaches (CHR18: 7 Mb and CHR15: 29 Mb) and all of them are novel. Furthermore, our results suggested that brucellosis infection is heritable (h^2^ = 0.26) and may respond well to genetic improvement programs. The GWAS analysis may support the development of markers to be used as useful adjunct in controlling the disease. In addition, new perspectives into the interaction between the infection and the diversity and composition of gut and vaginal were obtained.

### Supplementary Information

Supplementary Information 1.

Supplementary Information 2.

## Data Availability

All results are presented in supplementary files. The raw sequence reads of vaginal and fecal microbiome were deposited to the SRA at https://www.ncbi.nlm.nih.gov/sra/PRJNA910086.
